# Aspirations and realities in a North-South partnership for health promotion: lessons from a program to promote safe male circumcision in Botswana

**DOI:** 10.1186/s12992-016-0179-3

**Published:** 2016-07-28

**Authors:** Masego Katisi, Marguerite Daniel, Maurice B. Mittelmark

**Affiliations:** Department of Health Promotion and Development, University of Bergen, PO Box 7807, 5020 Bergen, Norway

**Keywords:** Safe male circumcision, Botswana, Partnership, Donors, Finance, Ownership, Target, Synergy, Antagony

## Abstract

**Background:**

International donors support the partnership between the Government of Botswana and two international organisations: U.S. Centers for Disease Control and Prevention and Africa Comprehensive HIV/AIDS Partnership to implement Voluntary Medical Male Circumcision with the target of circumcising 80 % of HIV negative men in 5 years. Botswana Government had started integration of the program into its health system when international partners brought in the Models for Optimizing Volume and Efficiency to strengthen delivery of the service and push the target. The objective of this paper is to use a systems model to establish how the functioning of the partnership on Safe Male Circumcision in Botswana contributed to the outcome.

**Methods:**

Data were collected using observations, focus group discussions and interviews. Thirty participants representing all three partners were observed in a 3-day meeting; followed by three rounds of in-depth interviews with five selected leading officers over 2 years and three focus group discussions.

**Results:**

Financial resources, “ownership” and the target influence the success or failure of partnerships. A combination of inputs by partners brought progress towards achieving set program goals. Although there were tensions between partners, they were working together in strategising to address some challenges of the partnership and implementation. Pressure to meet the expectations of the international donors caused tension and challenges between the in-country partners to the extent of Development Partners retreating and not pursuing the mission further.

**Conclusion:**

Target achievement, the link between financial contribution and ownership expectations caused antagonistic outcome. The paper contributes enlightenment that the functioning of the visible in-country partnership is significantly influenced by the less visible global context such as the target setters and donors.

## Background

### Partnerships for health

The Botswana Safe Male Circumcision (SMC) program is a North-South partnership aiming to promote sexual health via voluntary medical adult male circumcision [1, 2]. The Botswana SMC program was established to help meet a particular public health target – the prevention of HIV via the medical circumcision of 80 % of HIV negative men. As important as HIV prevention is, the research reported here is not about progress in meeting that target. Rather, this is a study of how the Botswana SMC program functions as a North-South partnership. Such research is urgently needed, because many North-South health partnerships function poorly, and they fail to meet their goals [3, 4]. By studying what factors promote and inhibit good partnership functioning in existing projects like the Botswana SMC program, the aim is to generate knowledge that may help future North-South health partnerships better meet their goals.

Medical male circumcision is recommended by the World Health Organization (WHO) for countries that have high prevalence of HIV infections and low practice of male circumcision [1, 2, 5]. Randomised control trials provide evidence that the removal of the foreskin reduces chances of men acquiring HIV through heterosexual relationships by 50–60 % [6–8]. In 2014, Botswana recorded an HIV prevalence rate of 25 % [9], and Botswana is therefore one of the countries in which male circumcision is a major public health goal.

Global health issues like HIV call for global solutions. In modern public health practice for HIV prevention as for other health priorities, countries are expected to contribute the resources and expertise at their disposal. This approach recognises that only by combining resources can global society hope to achieve significant public health improvements. But how should countries cooperate in health development initiatives? A discredited approach of the past is the North-South donor-recipient model, with well-endowed Northern donor countries paying the bills for development in the South, and therefore making the decisions [10–12]. Since the late 1980’s, the preferred approach is the North-South partnership model [12, 13]. Yet as reviewed below, effective North-South partnerships are difficult to mount and maintain, despite the best intentions of all parties.

A well-functioning partnership approach to development has become so important that all of aid development adopts the Paris Declaration’s partnership framework. The Paris Declaration was signed in 2005 by the development Ministers of well over 100 countries and the heads of key international development organisations, agreeing international standards for ethical partnership on several dimensions: ownership, mutual accountability, managing for results, alignment, and harmonization [14, 15]. Authentic partnership implies a joint commitment to long term interaction, shared responsibility for achievement, reciprocal obligation, equality, mutuality and balance of power [16]. The idea is that partners with common interests and diverse resources can create synergy if resources are pooled to achieve partners’ common vision.

### True partnership is difficult

Even if partnership is the preferred model of North-South collaboration, it has long been observed that true partnership is difficult to achieve and maintain when resources are unevenly distributed [17]. Power is unevenly distributed in most North-South partnerships [18], including AIDS prevention partnerships [19–21]. Northern and Southern partners may have very different ideas about the meaning of partnership, as the term is value-laden and has many possible meanings [22]. North-South cooperation that is genuinely meant by the North to be a partnership may be perceived by the South to function in the donor-recipient mode [23]. Scepticism about Northern motives is fed by findings that at least some Northern actors use the idea of partnership in a rhetorical or an instrumental way [24, 25]. Persistent North-South asymmetry and perceived Northern domination has been reported in the literature right from the beginning of the North-South health partnership experiment [4, 11, 26]. A typical irritation is Northern partners’ emphasis on Southern partners’ accountability, experienced by Southern partners as a stripping away of their managerial autonomy [12]. Manifestations of North-South power imbalance can be quite direct; in his study of a Dutch-Sri Lankan partnership, Fernando [27] cites a statement made by an obviously frustrated Sri Lankan NGO leader to the Dutch partners:

*“As long as we agree, you say that the money belongs to both of us. But the moment we disagree, you say that the money belongs to you” (* [27]*, p.1)*.

### Research on partnership processes: a public health priority

Given the difficulties of health partnerships generally, and North-South partnerships in particular, research on North-South partnership processes and functioning is a public health priority. In a recent review of the effectiveness of North-South partnerships, Kelly et al. [28] conclude that the quality and rigour of the evidence base is thin. They emphasise that research is needed especially at the level of individual partnerships and the bodies that facilitate them. Kelly et al. [28] point to the need for indicators and frameworks that address the benefits and values of the partnership model of cooperation. They also emphasise the need for research on pathways (processes) that lead to effective partnerships. Others have also called for more and better quality North-South partnership research, and extend Kelly et al’s critique and call for new research in several important ways. Yassi et al. [29] call for a ‘communities of practice’ research mentality whereby Northern partners seek multi-directional learning – how can North partners improve their own functioning? Murphy et al. [30] have described practical tools to help North-South partnerships study the ethics of collaboration, with a view to ensuring benefits to all partners. Holmarsdottir and colleagues [31] call for the practical use of conceptual frameworks of North-South partnership to provide guidance about partnership practices, and point to the need for a critical stance in the conduct (and study) of North-South partnerships, given the “paucity of empirical studies that have been undertaken to both documents and deconstruct the collaborative process…” ([31], p280-281). Corbin and Mittelmark [3] describe a systems approach to the study of partnership processes, and a systems analysis of a North-South AIDS prevention partnership has addressed a number of the points of critique mentioned above [32–34]. It is observed that the proposed mechanisms or principles for accountability formulated for partnership effectiveness in the Paris Declaration are experiencing challenges and therefore need addressing [14]. Below we discuss ownership; mutual accountability - that poses expectations on financial contribution by the South; managing for results – that is outcome focused [3, 4, 17, 24, 33, 35].

### Mechanisms for accountability in partnerships

Ownership of programs by the recipient countries is overemphasised by UN agencies as a way to work against the observed imbalance of power between the North and the South experienced over the past decades. Ownership was added to the definition of partnerships in the 2005 Paris Declaration for Aid Effectiveness, and since then the term has become a buzz word echoed from all stakeholders [20]. International donors attempt to put the concept of ownership into practice, for example, though efforts to ensure that donor efforts are aligned to fit the local administrative and strategic systems [36]; promote domestic funding, and refine conditions to measure the commitment of domestic Governments to increase budgets towards HIV/AIDS scale-up programs [21]. Such efforts to cultivate ownership by the Global South follow events from the United Nations General Assembly of 2011, where UNAIDS appealed for shared responsibility in terms of increasing long term domestic and international funding towards health scale up programs, emphasizing that recipient countries should be held accountable for rising domestic investments on health [21, 37].

Mutual accountability and transparency in the use of development resources is vital in aid partnerships ([14], p4). The level of financial contribution is seen as an indicator of the recipient country’s commitment to the development programs [21]. Many African countries, in spite of low levels of income, have attempted to increase their share of AIDS expenditure to show commitment and ownership [38]. Only Botswana and Namibia had spending levels on HIV/AIDS sufficiently high to cover their full program requirements in the year 2013 ([21]: p.e56). Regardless of these local efforts, international investment in HIV/AIDS interventions continues to increase [37]. Seckinelgin [39] critiques the idea of promoting funding as if it is the only way to succeed in health interventions. He argues that putting significant funding into ineffective intervention structures will not yield effective results within a given space of time. For example he critiques programs that do not encourage behaviour change in those infected or affected by HIV but emphasise only spending more money. His contention is that although increasing funding on HIV prevention programs is crucial, it does not change people’s behaviour automatically therefore the right mechanisms of program implementation remain the most important aspect for success [39]. Partnership promotes other components of ownership like the political environment, local strategies to implementation and cultural relevance [10, 24]. McFalls assesses current partnerships in aid as not genuine, but as only deceptive strategies to “legitimise” the domination of the powerful under the pretence of benevolence [25].

Managing for results calls for measuring progress and assessing results [15]. The Paris declaration is clear that reporting outcome of results is vital to measure success [15]. The global aid environment, and more generally, health and development initiatives use targets and indicators to map success. For example setting numerical targets for programs was the norm and requirement for implementation of MDGs. Even the global agenda post MDGs still emphasis that target setting is critical for tracking success [40]. The commission led by Waage and colleagues to analyse MDG 1–7 note that “The use of results based framework is regarded as one of the strengths of the MDGs, and has certainly appealed in an aid context with the desire of donors to see measurable returns on investment” ([35], p1000). Fuduka- Parr and colleagues [41] explain that targets are actually used to monitor progress, to reward or punish recipient country and policymakers. While UN agencies see the target approach as powerful, critics see progress in quantitative achievements of some goals but observe numerous gaps that hamper achievements. Targets and measures are not easily conceptualised by local implementers and this is largely associated with measurement, ownership and leadership [35]. Several critics observe that the quantitative, target oriented programs as well as measures used side-line other important objectives like equity and quality in reporting tools. This makes interventions focus on ‘doing things right’ rather than ‘doing the right things’ ([36], p153), and this can only be addressed through inclusion of qualitative measures [35, 41, 42]. Given such challenges on the implementation of the mechanisms for accountability the recommendation by Corbin and colleagues to study partnership functioning and processes needs to be considered [3].

There is little research that examines the functioning of these partnerships and their authenticity [11, 33]. There are a few studies that analyse the functioning of partnerships including Weiss et al., Jones and Barry, and Corbin et al. [3, 33, 43, 44]. In analysing partnership between the North and one organisation in the South, Corbin et al. [33] observed that there was sharing of power between the partners; Jones et al. [43] emphasise the importance of trust and good leadership as key to success in partnership functioning; Weiss et al. [44] established that leadership effectiveness was a key ingredient to partnership synergy while administration and management did not really show any significant contribution to positive functioning. Power imbalance is the common finding in most literature on partnerships in general, especially between North and South [11]. Mawdsley et al. [18] argue that partnerships in international health initiatives frequently involve, on the one hand, blurry ownership of health programs by the Global South and, on the other hand, pressure from donors to ensure their conditions, expectations and targets are met. More positively, partnerships have been documented that stimulate the Southern partners to develop and increase their competence in population and reproductive health [45]. In their analysis of partnerships Bailey and Dolan [46] find the good and the bad. While Southern partners benefit from skills brought in by the North, gain capital benefits like infrastructure development, and develop a greater voice in the process, capacity building is still seen as a one way flow from the north [46].

### The case

The case in this study is the Botswana Safe Male Circumcision program. In adapting VMMC, Botswana calls it Safe Male Circumcision (SMC) program. This research attempts to answer the call above (for analysing partnership functioning) by exploring the functioning of the partnership between the government of Botswana’s Ministry of Health (MH) and two international organisations: U.S. Centers for Disease Control and Prevention (CDC); and Africa Comprehensive HIV/AIDS Partnership (ACHAP) to implement Voluntary Medical Male Circumcision VMMC [1]. The WHO recommended to VMMC implementing countries that 80 % of HIV negative men be circumcised by 2016 in order to make a significant impact on the countries’ current infection rates [47]. The two international organisations have worked with Botswana Government in many HIV intervention programs for a long time now, hence the Government calls them Development Partners (DPs) [48]. Their long term partnership with Botswana is set out in Botswana’s National Strategic Framework for HIV and AIDS 2010–2016 (NSF) [48], a locally developed document that provides strategic direction on the national response to HIV/AIDS [48]*.* Behind these Development Partners are the unseen international donors: PEPFAR which funds CDC and Bill and Melinda Gates Foundation for ACHAP. Different authors in partnership list different dimensions of partnership functioning including partnership culture, administrative and management roles, leadership, professional expertise, financial resources and nonfinancial resources, challenges with partner involvement and challenges that are community related [33, 43, 44]. We captured different partner roles and resource contribution in the partnership for SMC in Botswana. See Table 1.

**Table 1:** Partner roles and resource contribution

Partner roles and resource contribution	Ministry of health MH	Development partner 1 CDC	Development partner 2 ACHAP	PEPFAR	Bill and Melinda gates foundation
Financial contribution/In country donor	✓	✓	✓		
Development Partner (in the country)		✓	✓		
Coordinator and owner of program	✓				
International donor				✓	✓
Provides scientific expertise and skills		✓			
Provides training of staff on surgery	✓	✓	✓		
Provides training of staff on demand creation		✓	✓		
Provider of implementation staff for surgery	✓	✓	✓		
Provider of general medical equipment	✓				
Provides MC surgery kits		✓			
Marketing and advertisement of MOVE (large scale)		✓			
Provider of staff for mobilisation (Demand Creation)		✓	✓		
Provider of infrastructure nationally	✓				

This paper contributes to the scanty literature on the functioning of partnerships between the North and the South. The aim of the paper is to use the Bergen Model of Collaborative Functioning (BMCF) to explore achievements and challenges of the partnership on a Safe Male Circumcision (SMC) program in Botswana, while making efforts to attain the set target. Specifically we establish how the mission and functioning of the partnership contributed to the actual outcome.

### Conceptual framework

#### The Bergen Model of Collaborative Functioning (BMCF)

**Fig. 1:**
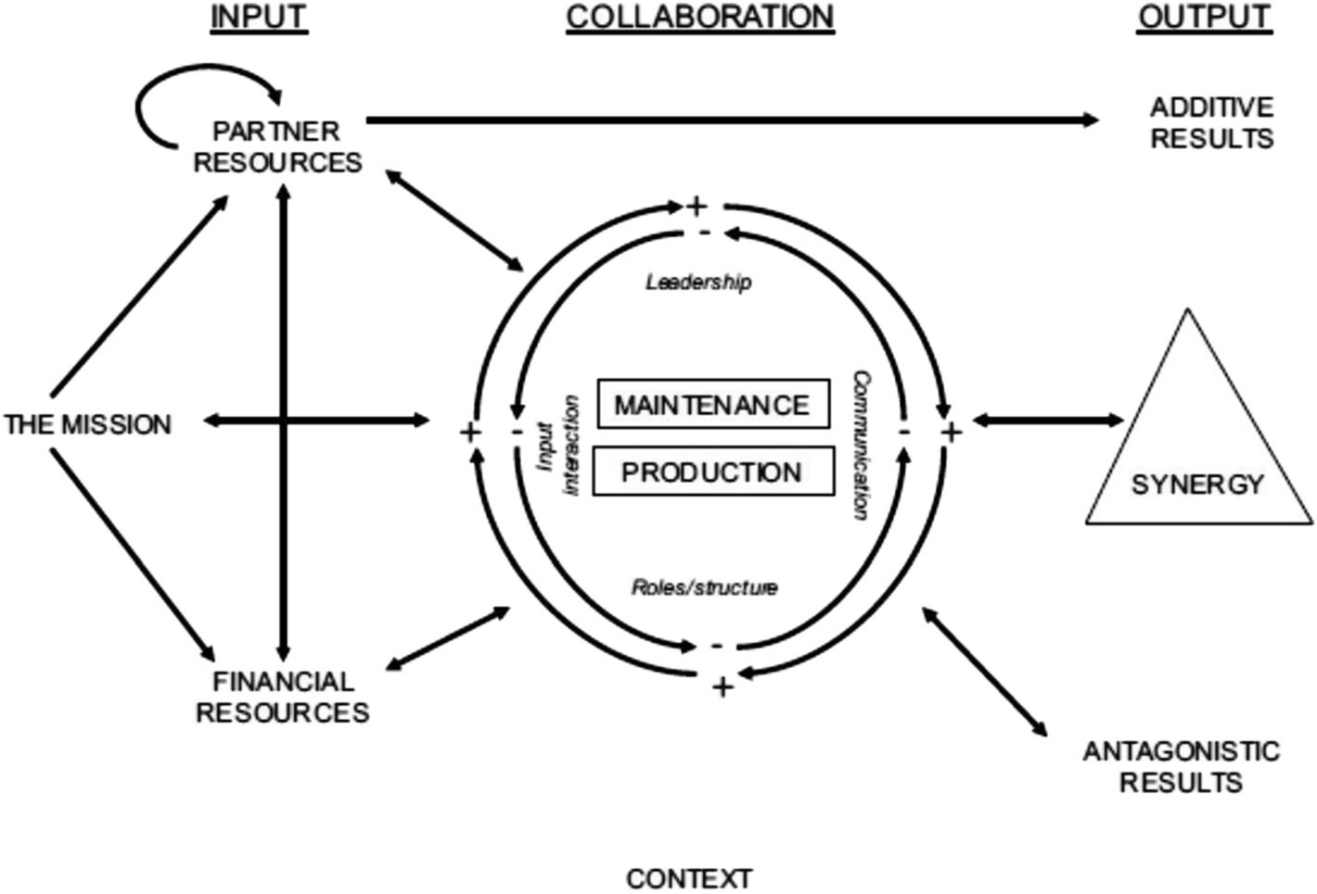
The Bergen Model of Collaborative Functioning: Adapted from Corbin JH, Mittelmark MB, Lie GT. Grassroots volunteers in context: rewarding and adverse experiences of local women working on HIV and AIDS in Kilimanjaro, Tanzania. Global health promotion. 2015:1757975915569514

We use a systems model, the Bergen Model of Collaborative Functioning (BMCF) [3] as a framework to examine the operationalisation of the SMC partnership in Botswana. The BMCF is shown in Fig. 1. We choose to use this model because it has been used before in assessing an HIV/AIDS partnership in the global South, [3, 32–34] and also that it addresses functioning. The BMCF model is useful in illustrating the contextual process within the partnership [34]. Inputs include the partnership’s mission (selected approach to deal with a problem), partner resources (knowledge, skills, competence, etc.) and financial resources (funding and material inputs) [33]. The collaborative context (or throughput section) of the partnership is analysed through the interaction – positive or negative – of four aspects that impact the maintenance (administrative) tasks and production tasks (related to partnership’s mission), namely, leadership, communication, roles & structure and the inputs themselves [33].

The output of the partnership can be *additive results* (unaffected by collaboration of the partners); or *synergy*, where more is produced by collaborating than if the partners had not interacted with one another; or *antagonistic results*, where the costs of partnership exceed the benefits [3, 33]. Additive results are things that could still be achieved without the partnership. This is based on the argument that a partnership is always built to tackle an existing problem where some action has been taken, anyway. Synergy is what is achieved because of the “multiplicative interaction” of the partnership. Put in mathematical terms it is 2 + 2 = 5 [33]. Determinants of synergy include partner relationship ingredients like trust and power, partnership assets, partnership characteristics, and leadership ([43]: p. 409). If synergy is achieved this may have a positive feedback impact on partnership inputs and functioning [33]. Reflecting on antagonistic results may also result in positive feedback [33]. Corbin and Mittelmark [3] note that it is possible for a partnership to include both synergistic and antagonistic elements concurrently. Corbin and Mittelmark’s theoretical contribution to the BMCF is the enlightenment on how context, specifically cultural and societal context, as well as partnership processes and partner contributions interact both positively and negatively to influence partnership functioning [34]. In assessing the volunteers’ participation in the organisation, they identified that positive results are generated by: the experience of social connectedness; seeing volunteering as opportunity for public recognition and for expressing passion to help others [34].

### The Bergen model of collaborative functioning: Fig. 1 should fit here

Corbin and Mittelmark [3] record that nearly 50 % of partnerships dissolve early and impulsively. They observe that resources, characteristics of partners, features of the partnership strategy and environmental factors can either support synergy or create antagony between partners ([3]: p.365). Fowler [4] adds other elements that may cause antagonistic outcomes in partnerships such as: paternalistic behaviour of those with cash power; upholding the approach of the Northern rather than the Southern partners; hiring staff from the North because of capacity limitations of workers from the South, and the North’s anxiety about loss of control. Corbin and Mittelmark [3] argue that although more financial resources can improve the functioning of the relationship, funding can also complicate functioning: antagony is created if partners and funders view the partnership as a waste of financial resources and time. A study in Indonesia found imbalance in the governance of the partnership, adapting a top down approach, where priority areas were defined by the multilateral agencies only and were a reflection of their own concern in the governance field, not those of the local people [24].

## Methods

Given the dynamism of partnerships and our intention to explore views of administrative and implementing officers at different levels we chose qualitative methods to achieve a broad and diverse understanding of the working of partnerships in the SMC program in Botswana.

### Research sites

There were three research sites based on the location of the different partners at national and district level. National Leading officers for the major partners, MH and DPs were in Gaborone, the capital city. Local implementing/administrative officers, District Health Management Teams DHMTs were in the other two sites, Mochudi and Hukuntsi villages. These villages were purposefully selected as two villages with contrasting population and geographic sites: the former being highly populated and close to the city where services are easily accessed; and the latter being sparsely populated and in one of the remote areas of the country.

### Participants and recruitment

Groups of participants from national to districts level took part in the study. Although participants at national and district level were not promised anonymity of their organisations, for confidentiality reasons we refer to MH partners as DP1 and DP2. Participants comprised leading officers working for the three partner organizations, MH and its DHMTs, DP1 and DP2. The SMC lead officer in MH introduced the first author to all the partners through a 3 day annual planning and strategy meeting that she was invited to attend in 2012. Observation in the meeting created a platform for the first author to establish rapport, enabling direct contact with leading officers from partner organisations to set interview times. DHMT representatives that attended the 3-day meeting were not gatekeepers at district level; therefore the higher officers at the DHMTs were approached directly using permission from MH. In that way access to the district health officers was granted. The first author had personal links to both communities, which made it easy to access the participants.

### Data collection

The first author spent approximately 18 months in Botswana between December 2012 and August 2015 collecting data and observing the different phases of the SMC partnership. The research question set out to explore the functioning and contextual interaction of the SMC partnership in Botswana. The research used three qualitative research methods: observation, one-on-one interviews and focus group discussions (FDG). Non-participant observation was used during the partners’ 2012 planning meeting since the researcher was not allowed to comment but just listen and take notes. Follow-ups were only allowed outside the meeting room where the researcher could have informal conversations with the participants. The 2012 planning meeting informed the research on the fundamental themes to explore within the parameters of the research questions. Data from the meeting influenced the direction of the data collection for the rest of the research project: who to ask what, and where, and also guided the drawing of the interview and FGD guide, as well as the observations guide (adjustments made on the ground). This included topic guides and questions on: the mission of the partnership; leadership of the partnership, partners’ resource contribution; partners’ roles; general functioning of the partnership and of SMC implementation.

Data were collected through a 3 year period and with great attempt to do it in a cyclic manner (with follow-ups of the same key informants where possible); and following important incidences in the partnership processes. We used an observation guide at the 2012 partners’ planning meeting where 30 participants from all three organisations gathered, interview guides for rounds of interviews with key national officers leading the program within each organisation (three lead officers at MH and one from each of the DPs). A total of eight interviews were conducted with the national officers, including three follow-up interviews with one lead MH officer over the years. Three FGDs comprising five to nine participants were carried out with the Mochudi and Hukuntsi DHMT teams respectively, as well as the Mochudi MOVE team. For interviews and FGDs we developed a semi-structured topic guide. We asked questions like; What is the mission of the partnership? What roles do different partners play? What resources do partners contribute?

The range of data collection methods employed generated diverse data and was also a good source of triangulation, linking what is discussed at national level with what took place at district level and on the ground. All data were collected from December 2012–2015. Data for all three parts of the BMFC theoretical framework, inputs, throughputs and output were covered throughout the period of data collection in a progressive way. For example there were new resources contributed or withdrawn at different times, repetitive activities run throughout the years, negative and positive outputs realised at different phases of the partnership.

### Data analysis

The observation session for the 3-days-review and planning meeting was recorded as detailed written notes following the preference of the participants. All other interviews and focus group discussions were audio recorded with permission from the participants. Three research assistants transcribed the data. In order to ensure accuracy and validate what was transcribed the first author translated all transcribed data from Setswana to English and cross checked those already transcribed in English to verify transcripts against audio recordings. NVivo ten qualitative data management computer software was used to manage and analyse data, hence all data were imported into the software for coding using Attride-Stirling’s Thematic Network Analysis [49]. The data analysing team comprised two PhD students as well as the first and second author. The various data sources were analysed connecting different groups of participants. Following Attride-Stirling’s [49] stages of data analysis, data were initially read to code topics raised by the participants. Then basic themes were abstracted from the coded sections and grouped into organizing themes, which were then further clustered to global themes. In view of these themes, we reflected on our data to get an understanding of the working of the partnership in question, relating our findings to our research question. In order to strengthen the objectivity of the analysis, the team discussed organizing and global themes to reach a consensus throughout the analysis, ensuring stability and relevance.

## Results

The findings are presented according to the BMCF as our theoretical framework. Four elements of the model being: 1. Input, 2. Throughput and 3. Output and 4. Feedback to mission, form our global themes. Basic and organising themes that emerged from the analysis are presented in Table 2.

**Table 2:** Themes emerging form data analysis: Applying the Bergen Model of Collaborative Functioning

Basic themes	Organising themes	Global themes
1. Botswana government HIV National Strategic Framework (NSF) lead by NACA 2. All ministries, development partners CBOs, NGOs and private sector are part of the NSF 3. MH, DP1,DP2 are three main partners in the SMC program 4. DHMT works at district level 5. All partners involved throughout the planning process since 2007 6. All partners target HIV negative men aged 15–49 years to circumcise through SMC 7. All partners aim to have circumcised 80 % of HIV negative men by year 2016	Clear Partner Mission	Input
8. Botswana government integrated circumcision within health services nationwide since 2007 9. DPs introduced MOVE project in 2011 to help government push set target in selected areas	Approaches to the mission	
10. DP1 viewed as a major financial contributor: more monetary funds; sub-constructs companies; built 2 permanent clinics; provides surgery kit; provides mobile clinics and transport 11. DP1 contributes funds and funds medical personnel and transport 12. MH contributes funds; provides health structures nationally; provides medical equipment and transport	Financial Resource Contribution	
13. DPs deployed medical staff to Government health centers to do SMC 14. DPs deployed staff moved to form dedicated MOVE teams 15. DP1 brings in special scientific expertise 16. MH’s avails its medical staff nationally to participate in SMC	Partner Resource Contribution	
17. MH as owner, coordinator, chair, provider of space and financing 18. DP1 as technical advisor, expert, advertising, mobilisation, provider of clinics structures and main donor 19. DP2 as donor, implementer and community mobiliser	Clear Partner Roles	Throughput
20. Partners developed short term and long term communication strategies; training manuals and reporting system together	Communication	
21. Development partners use different reporting systems than MH’s 22. Development partners do not report to MH systematically 23. Reporting between partners was not transparent 24. Development partners reported directly to their international donors 25. The Government reported all donor funds usage to OECD 26. Way of accountability give blurry structure		
*Financial resources* 27. More finances spent but less numbers of circumcised men causes conflict 28. MH’s financial contribution queried to be not transparent 29. Ownership seems linked to finance contribution 30. MH’s ownership of the program is questioned 31. MH sees structures as big contribution	Input Interaction	
*In-kind resources* 32. Donors keep sending more equipment for circumcision 33. Lots of equipment is wasted 34. There is inconsistency on balance sheet for number of circumcision instruments, wasted and remaining 35. MH is blamed for not taking care of such equipment		
*Partner resources* 36. MH viewed as a weak coordinator at times 37. MH ownership is queried 38. Government health centers is blamed to be participating little in circumcision 39. MH feels MOVE strategy naturally creates dependency on government health staff 40. Districts prioritised attending to ill patients than circumcision 41. DHMTs blamed for not prioritising SMC 42. Health centers viewed SMC as the DPs’ program		
43. Partners consulted with the national traditional leadership at planning stage 44. MH is seen as a leader and owner 45. There is not enough support from the highest national leadership to influence men for circumcision 46. MH’s placements of coordination leadership is queried 47. DHMTs are said to not take leadership role accordingly	Leadership	
48. DPs blame MH for setting the target high 49. DPs blame MH for not putting enough effort and resources to push the set target 50. MH is frustrated about the mathematical model used by WHO to set country target 49. Unattainable target is seen as the highest risk in program implementation 51. MH and DPs express frustration that the 80 % target is not attained regardless of their massive efforts 51. DPs report pressure from donors on reconciling dollar to numbers	Mission threatened	Feedback mission
52. DPs indicate that the donors will cut down on the funds 53. International donors reduce funding support to Botswana		
54. DP2 pulls away its employed doctors gradually from 2013 and leaves a gap in implementation 55. DP1 pulls away its financial and technical assistance abruptly in 2014 and leaves a gap in implementation	Antagony	Output

### Input

#### The partners’ mission

The Government of Botswana has an established national strategy and a framework of operation for all health initiatives, called the National Strategic Framework (NSF) that is reviewed every 4 years. The NSF comprises all ministries, with MH being the lead ministry; long term development partners like DP1 and DP2; civil society organisations and NGOs; and the private sector. The same development partners were partners in the SMC program. Several officers explained this. An example follows:

*We, the DPs are always in the country. We are here to help with all HIV/AIDS intervention strategies.* (Lead officer 5 in Gaborone, during the second round interview*).*

All partners were clear about their mission: to get HIV negative men of ages 13–49 circumcised in order to reduce HIV infection rate in the country. They were also all working towards a target of circumcising 80 % of HIV negative men by the year 2016, which is 100 000 men in a year. They divided the target between them. The partners were also clear about their commitment to the mission. One DP1 participant explained their commitment towards achieving the mission:

*So we have at best 40 % of the 100 thousand target that is to be covered. At best 40 % of the target is our aim as DP1*.

One DP2 officer at the 3-day meeting added:

*We aim for 25 % of the target…*

Although the MH did not state a percentage they were aiming for within the 80 % target, they explained that their aim was to integrate SMC in the health system nationwide, establishing it as a long term program, not just up to year 2016. The 80 % target by 2016 was defined as a project within the Government’s long term program.

#### Approaches to the mission

There were two approaches used in implementing SMC in Botswana: The integration of SMC in the whole health system that was locally planned and designed; and the MOVE approach that was externally introduced through DP1, and adopted in 2012 in parallel to integration. At the beginning of SMC implementation between 2007 and 2009 all partners worked together to develop and implement integrating SMC in the whole health system. Lead officer 3 explained:

*Government’s long term plan is to integrate SMC into the normal health system. We worked on this with our partners from the beginning.*

Lead officer 1 in round 2 interview explained the integration target per health facility:

*We expect clinics to circumcise one client per day or 5 clients per kilometre through our integration strategy.*

The integration strategy did not circumcise enough men to approach the target yet MH had an obligation to meet target by 2016. The new MOVE project was introduced by DP1 in 2011 to help push target. All partners embraced it and MH viewed it as great help. Lead officer 3 explained:

*…..but then this idea of the MOVE project came in in 2011….It was introduced by PEPFAR through DP1. Through move MC is marketed and advertised to get many numbers of men to circumcise at the same time.*

Lead Officer 1 also expressed appreciation of MOVE:

*The Government is not enough alone. We are weak alone…you see? We welcome the development partners to fill in the gaps…, you see… With targets set, Government needs assistance…. We appreciate the MOVE project because there is a lot of good in it; to help us reach the numbers.*

However, MOVE was not covering the whole country so issues of equity were of concern to the MH: In giving the official opening speech at the 3-day meeting, an invited high official commented on the need for demand creation of the program nationwide. He said:

*Government emphasises equity. We need to strive for equity, not just a few districts for the MOVE project but all districts. Circumcision, circumcision, circumcision is our breath.*

#### Resources

Resources include financial, capital and staff resources. MH appreciated partner resource contribution in both the integration and MOVE implementation strategies. Lead officer 1 explained:

*Because of challenges of resources we thought the idea of combining resources with DPs was a good one.*

#### Financial resources

The DPs contributed financial resources towards achieving the mission. The MH also contributed its national funds and mobilised other international funding support besides DP1 and DP2 partner contributions. Although it was not possible for the first author to access partners’ contract agreements, it was communicated clearly that for MOVE implementation, the DPs contributed massive monetary resources to help push target. DP1 was said to be the main financial contributor. The impact of resource contribution by development partners was experienced even at implementation level. One DHMT officer stated this in an FGD:

*Many people turn up for circumcision when the MOVE teams from the DP1 contractors come. They come with lots of resources you see…last time they were here they brought vehicles which were used to fetch people from settlements around to come here in the hospital for circumcision. They also have lots of staff. We don’t, we are overwhelmed with many other duties. Not just circumcision.*

#### Partner resources (skills)

Partner resources include skills and other in-kind resources other than money. The MH availed all its heath facilities and medical equipment in the whole country to do circumcision. The health facilities and some of the medical equipment were used by the MOVE dedicated teams as well. DP1 provided mobile clinics and constructed two main permanent clinics in the capital city; and brought in 80 Peacecorp Volunteers specifically to push the SMC target. At the time of the data collection in 2012 they were promising ‘clinics in the box,’ fully equipped mobile trucks. DP2 also gave funding, provided about 30 foreign medical doctors and a number of nurses to do the surgery, and paid community mobilisers to recruit men for circumcision. Lead officer 4 explained further on DP1 contribution:

*So what we bring to the table as DP1 is a level of scientific knowledge that many organisations don’t have….Prepex study… but we also bring in experience from other countries on how partnerships work and how coordination can occur and how systems can be built…monitoring and implementing change…that kind of a thing.*

The DPs supported the integration program by deploying staff to government health centers around the country. One of the MH officers explained:

*When we started the integration program, the DPs deployed their staff (seconded doctors and nurses) to government health centers to work with our nurses and doctors in circumcision.*

When MOVE implementation started more doctors were employed by the DPs to form dedicated teams for both static and mobile clinics. Lead officer 1 explained this:

*When they promised dedicated MOVE teams and we know that in Botswana we have skeletal staff…..just the few of us, why would we refuse?*

Additionally, DP1 outsourced contractors, some to do training, one to do marketing and demand creation and others to carry out the surgery. This appeared to be the main difference between integration and MOVE. One MH officer explained:

*When the MOVE idea came in it overpowered the original one. In MOVE, DPs brought in demand creation strategies like adverts on TV and radio, public campaigns, mobile clinics for circumcision and staff to do the job.. So we cover many people at a time. Integration is a long term program and is not as fast as MOVE.*

Lead officer 5 explained further on their contribution to bring speed to the project:

*We serve as catalysts to government..basically making it do things faster because we are always focusing on cost effectiveness. We have brought in 30 medical doctors to help move target.*

Lead officer 5 added:

*We provided 30 doctors to train other staff and to do surgery in dedicated clinics.*

The government staff that was already employed continued to participate in SMC through the integrated program with the health system.

#### Throughput

The context for partnership operation comprises maintenance/administrative tasks which in this case included development of communication strategies, training and reporting systems; and production task which are implementation activities like periodic funds injection, equipment purchases, demand creation activities and the surgery (circumcision). As the inputs interact during production and maintenance activities through time, roles and power struggles are manifested. This is shaped by the interaction of roles, input, leadership and communication. There can be both positive and negative experiences as the partners interact to work together. We therefore present such interaction, some of which overlap.

#### Clear partner roles (roles/structure)

All officers interviewed defined the partners’ roles the same way: MH as owner, coordinator, provider of structures and equipment, staff for the integration program and financing; DP1 as an in-country donor, technical advisor, expertise provider, advertising, mobilisation, provider of clinics structures; DP2 as an in country donor, organisation and provider of implementing staff for the MOVE program. Although there was manifestation of power struggle during maintenance and production activities, the roles did not change, for example government still maintained its role as the custodian and owner of the program. See Table 1 for roles.

#### Communication

Strategic documents were developed together at national level. All partner representatives explained that they had worked together as partners from the inception of the program in terms of strategizing on the implementation approach and developing strategic documents: communication strategies, monitoring and evaluation plan 2010–2016 and the reporting systems. Lead officer 1explained this:

*Year 2007–2008 was a planning period. As the other presenter stated all operational documents were developed then. We formulated these together….The integration of SMC in all health centres started in 2009.*

Lead officer 1 made the hierarchy of the partnership clear:

*We, MH, started the SMC project as “parental states”*^1^*but with the partners participating. We started with trainings, formulation of strategies and so on. Development Partners have always taken part in SMC from the beginning with government leading.*

Another of the DP2 participating officers clarified that the documents were developed in consultation with the traditional leadership at national level. He said:

*Remember we got guidance from the House of Chiefs on what circumcision is called in Setswana* [Botswana national language]. *We tried to engage those who could help in proper language.*

The government clinicians on the ground were also involved early in the program. They took part in developing the SMC curriculum. One officer representing DP1 explained as he argued that doctors need to be involved more:

*So you see…, when we were developing the curriculum that time we were getting a lot of input from the clinicians as to how we can improve it.*

Whereas at planning stage the partners agreed on the same reporting systems and communication strategies, there seemed to be divisions and differences at implementation level. There were queries that the DPs were using their own separate manuals for implementation and own reporting system different from the initial ones formulated. One officer queried this during the 3-day meeting:

*We need to fast track the issue of the different training manuals so that we have a document that is standardized. The manuals between MH and DPs have differences here and there…. we need something standardised.*

MH further explained that the DPs were not reporting consistently to them as the coordinating organisation in the partnership. DPs admitted that they were using different reporting systems.

### Input interaction

#### Partner resources (staff)

The partners discussed and worked on strategies for working together. Staff resources were a challenge to government, therefore they appreciated that partners could provide staff to support the integration program. Lead officer 1 said:

*Our districts had a challenge to take circumcision in at a massive scale. So it was good for us that the DPs seconded their staff to the health centers.*

However, the deployed DP staff queried that they were assigned other duties in government health centers and this interfered with strengthening the SMC program. It seems the MOVE project helped address these queries and maximised focus on the mission.

Lead officer 5 explained how they solved the problem:

*Forming dedicated teams was the best arrangement for the MOVE project so that we can focus and push target.*

Even though the MOVE teams were separated to work alone, resources continued to be shared to support the mission. Government supported MOVE teams with vehicles and other medical equipment. However, during FGDs all DHMT staff expressed that there is continuous tension between MOVE staff and government health management staff on provision of resources. One said:

*You know the MOVE team sometimes needs vehicles for mobilisation of other activities. But we cannot always provide them with vehicles. Most of the time our transport is committed to other health duties, transporting sick patients…and then we are seen as not supportive of SMC.*

DPs also complained about lack of commitment by DHMT staff, which they called “dependency.” The “dependency” seemed to be caused by the fact that the health personnel in the clinics did not regard SMC as a “priority” program compared to ailing patients. Several officers have reiterated this throughout the years, both at national and DHMT levels. One said:

*SMC is not a priority within Government clinics. When there is a diarrhoea outbreak or a bleeding patient or something, that is what is given attention. SMC clients are made to wait or return.*

Another said:

*If you go to the district these days the districts are not seeing SMC as anything.*

Following up this issue at district level with the DHMTs, many expressed the same thing in both group discussions and FGDs. An example follows:

*You see, it is not that we are not taking SMC serious. This is a prevention program. But sick patients are a priority to us. Also, we are understaffed in clinics and so we have to prioritise…but we try.*

A DP1 officer added:

*There is evidence that when DHMT coordination is leading and participating, things move, but when it is not there, little moves. We need a way to make DHMT own the program.*

DPs also questioned the placement of regional coordinators within MH, suggesting that the north coordinator’s office should move to the north. However MH argued that this would not work efficiently for national coordination. One MH officer responded to this:

*This is not the first time I hear of this suggestion. You wouldn’t be happy if I do that to your office.*

There was no query on the DPs’ staff performance. However, some DPs staff roles were not clear to MH. When one of the leading officers in MH was asked about *Peace-Corp* volunteers he answered:

*About Peace-Corp I do not know what they are doing. I really don’t know if they can make any impact when Batswana youth are failing.*

A year following these interviews, the *Peace-Corp* volunteers had stopped working for SMC. Although none of the officers at national or district level made any comment about the MOVE team doctors, they were all foreign employees.

#### Financial and technical resources

MH appreciated the massive financial resource contribution by DPs regardless of reported delays to fulfil promised funds on time. However the high level of funding gave the partner a wedge to question issues of ownership, defining a blurred relationship between partners. DPs queried that they contributed more money to SMC than government and that they were transparent about what they gave but MH was not. Lead officer 5 queried lack of transparency from MH:

*The Government is supposed to govern..,. You see now we say DP1 brings so much money to the program, DP2 has so much money… The Government keeps quite, that’s why we are asking can the Government tell us what its budget is… This is one question I have never gotten an answer for.*

The conversation below between two officers at the 3-day meeting shows more questioning on government’s level of contribution of financial resources.

Lead officer 1:

*So, in terms of funding circumcision, the Government of Botswana has money. The Government creates its funding pot from all over, I cannot exhaust the list. For example 19 million pula is projected to come from (an international organisation mentioned).*

Lead officer 4:

*How does Government make its plan then? Is it donors first, then Government.*

Lead officer 1:

*You are supporting me. Don’t ask me what I have, bring what you have….The Government provides all clinic structures, we provide staff, and we provide equipment.*

MH further explained that besides external support government gives out funds from its own internal budget. MH also asked the meeting participants to be aware that government has offered infrastructure, equipment and its medical staff for implementation of SMC. Contrary to resources as key to SMC implementation, several officers from DHMTs reported that even where MOVE brought lots of resources, men still showed resistance. This was explained more even in later interviews (round 2 and 3) and also witnessed at the MOVE campaigns. One DHMT officer said:

*I do not think the main issue should be resources only as you see it. You know even during large MOVE campaigns here in Francistown* [second largest city] *where we have all resources in place men come to listen in quite large numbers we still have a few turning up to circumcise.*

Whereas DPs felt they were using more resources yet getting poor results and whereas there were few numbers of men circumcising than expected, external donors still spent more money on supplying circumcision equipment (kits). In her presentation, one officer from MH reported that there was wastage of equipment in the clinics because the numbers turning up for circumcision does not match the massive number of equipment purchased. She reported:

*…78 000 kits were bought and given out; 33 000 kits were used therefore 45 000 remain. But only 25 000 were accounted for… This means 20 000 kits are missing or wasted. Hundreds of kits are sent to districts yet only a few SMCs are done in a month. We overestimated numbers.*

Lead officer 4 responded to this presentation with concern:

*This exact issue is the same thing we are going to be nailed about as we account to the ambassador and to Washington DC.*

#### Blurred roles and structure

Whereas MH was not managing partners’ funds and transparency between partners on utilisation of such funds was limited, the MH still had to account for all funds as received country funds to the program. In this way, accountability seemed to be in one direction. MH officer 1 explained the dilemma that the Government faces when reporting OECD, having to act as a parent who protects the partnership:

*When the high office in MH reports to OECD he reports all funding as the Botswana basket. He cannot tell them that most of the donor funds are spent on overheads, paying contracted companies and administration, not on the client, even though this may be true. He has to speak like a parent, in a way that would bring more support in the future.*

The Government has to account for funds without knowing the details of the DPs budgets.

*It is not easy to ask “how much are you paying your Coordinator?” It is an internal thing. That is how it is and that is the life we have to live…We have to account and have to ensure that we are not blamed.*

#### Leadership

In the 3-day meeting, there was a reflection that the partners appreciated the administrative leadership of the program (officers leading). However, there were queries especially on MH as the owner, host and leader of the program. This is presented under resources above. Ownership is also understood in terms of commitment. During the meeting and the round of interviews, development partners expressed that the levels of commitment to the program differ within the MH’s different levels; with high commitment at national level but little commitment at district level. Meanwhile the MH viewed these as teething problems of a new program: Lead officer 2 explained:

*..with a new program you will always experience a challenge in the first 2 years of implementation.*

Another officer saw it as a common problem on vertical programs. During interview 2 in Gaborone, Lead officer 3 said:

*All health programs start as vertical programs and have challenges, but the aim is to see to it that these programs are integrated within the existing system.*

In addition, country leadership is considered important in international partnerships. There were comments of appreciation for the support from some of the Botswana political leaders in high positions. One Member of Parliament was regarded as a champion because he circumcised under SMC and was campaigning for the program in his constituency. Some politicians at community level were also cited as supporting the program. However, commitment of the country leadership was questioned by all three partners. There was a conversation on this issue among all the three partners. One said:

*But in my opinion have you ever seen the high leadership of this country coming out and saying “citizens of Botswana lets circumcise.”*

The house laughed at this comment. Lead officer 3 responded:

*Do not mention higher leadership when talking about this program.*

The same officer shared that the higher office at MH is working on getting the influence of the national leadership. Another officer his experience with Members of Parliament:

*The committee we met at parliament said “Our hands are full, consult with the community. Whatever the chiefs say is what the communities do…”*

It seemed that consultation with the high political leadership was done superficially. One officer shared feedback from the House of Chiefs:

*We also talked to the House of Chiefs and they complained that they are not being involved in the program. They said they needed adequate information to articulate issues that are to be addressed.*

Although there was contention on resources and the target partners made an effort to find solutions to the problems. For example, during the 3-day meeting they divided into several groups to discuss how to address the challenge of high ‘unachievable’ target; different demand creation strategies to recruit more men; how to strengthen DHMTs to be more involved in SMC. Financial tensions were not addressed in the groups. However, the cyclic interviews following the meeting (in 2013 to 2014) not reveal much implementation of the brainstormed ideas.

#### Feedback on the mission

Feedback on the mission was experienced at different phases of the partnership. As partners met annually they reviewed the mission and strategized on improving their approaches to implementation. However, pressure from donors resulted in the DPs retreating. During the 3-day strategic meeting, the participants assessed risks to the program goal achievement. The 80 % set target was the first thing to be listed as a risk. There were debates around this target with questions as to why MH accepted it while it was not realistic to Botswana setting. It was explained that WHO used mathematical models to calculate the target for Botswana. A sample conversation on the target follows:

Officer 1: *There was pressure of 100 000 not being negotiable or debatable… If these directives are not realistic they have to correct themselves on the way. You cannot achieve by just demanding the plan.*

Officer 2: *Yaah we were being forced to achieve something that is not achievable.*

Officer 1: *…there is an option that we could increase the number of years but maintain the target but this will affect impact. Although the targets are not negotiable, they cannot be reached…. It’s a risk*.

*Officer 3: It’s even worse that we did not even achieve 50 %… The DP1 target was 30 000 but we met only 9 000.*

Nonetheless, partners appreciated their efforts in working on the mission together. They appreciated that the pressure was really from beyond themselves. Lead officer 5 commented:

*My comrades at MH… we are all really doing the best we possibly can but we are being pressurized from above by our superiors.*

The target still had to be reached according to WHO requirements and external donors’ expectations. The pressure of expectations fuelled the tension regardless of the partners understanding of the target being unrealistic. There were several complaints from the development partners that MH was not taking the lead in implementation speed as the owner of the program. The integration strategy was blamed to be slow. One officer from DP1 queried:

….*In the integrated sites where there are no development partners, the performance is down.*

This was also revealed in the MH statistical report during presentations at the meeting. The integrated sites had attained just over 4700 men between 2011 and 2012 while the MOVE sites reached over 37,000 men in the same period. Hence 100,000 men were not reached. Whereas partners blamed this on MH for not putting in more financial resources the officers at implementation level had a different view that there were other individualised and community or peer collective reasons why men were not coming for SMC. They appealed that these community reasons should be considered.

### The mission threatened

In 2012, the development partners were already expressing possibilities of withdrawing from the program because of low target achievement. Although MH claimed ownership; it felt like a weakling when partners mentioned possibilities of withdrawing their services. Lead officer 1 expressed:

*You can’t wean a baby overnight. You can’t do it just like that. It should be a process.*

Lead officer 4 then promised:

*We are not weaning the baby; we are not weaning Government now. So do not worry.*

Lead officer 4 added later in interview round 2:

*We certainly know one of our competitive advantages is money, and that’s great but you can throw money out to a problem and it does not solve a problem, which we are learning and that is why we are opting to stop if we are not successful.*

#### The mission unaccomplished - Partners compelled to pull out (Year 2014)

The DPs ultimately ceased their services, DP2 withdrew slowly from 2013 till February 2014 and DP1 later in 2014.

The Government seemed frustrated when development partners ceased activities. At the same time it empathised and expressed appreciation of why they pulled away. Lead officer 1 explained in round 3 interview in 2014:

*Yeah I mean we are required to account for every dollar used. They would calculate a certain number of circumcisions to dollars….so the development partners have to account for this….. We reached only about 39 % of the set target in 2012……. It is not their choice to be pulling out. They have pressure from the donors… the donor pulled away… They feel their funds are not used efficiently.*

The departure of the DPs set back the performance of the program to the time it started, without the MOVE project. Additionally this called for more expenses from MH to cover the gaps created by MOVE. The same officer explained:

*Since DP1 pulled out men are coming to the facilities for circumcision but there is not enough staff there to circumcise them. So we are back to square one. We are experiencing the very slow numbers we were experiencing when we started… Government Development partners pulled out from dedicated clinics. Now it is expensive for Government.*

#### The mission revisited -MH revisits the SMC strategy

The MH seemed ready to face the reality of true consultation with communities alone. The officer explained:

*We are now back to the basics to tell the truth. We have lost the support of partners as a country. But we are not going to sit back and say VMMC is not possible in Botswana. We are not giving up…We are thinking of addressing these basic issues. Like this year we really want each district to ensure that we are involving the local authorities…*

The officer confessed that they missed important community consultation from the beginning*:*

*We really missed it. We missed the behavioural issues… and the cultural issues. We should be one in this issue with the tribal leaders such that when I leave here and go to Ramotswa, the chief should not see me as Ministry of Health, but as one with his community, to help the community…..they should be saying we are in this together*…

Although it was not carried out, the development partners had also vocalised the need for true consultation with communities before they pulled away: One of the officers from the 3-day meeting had earlier expressed:

*We need some kind of synergy between what the SMC does and the traditional practices out there.*

Lead officer 1 mentioned deploying youth as one of the issues to be revisited:

*..when you talk about demand creation, we have learnt lessons from MOVE. We now know that we cannot use young people to talk to older men. It doesn’t work.*

Other issues raised during discussions on why the target was not reached concern men’s fear of pain, peer influences, and the need for women involvement.

### Output

#### Additive results

Additive results are things that could have still happened without the partnership. In the findings MH revealed that circumcision had always been part of the services of the Botswana health system, just not offered at a massive scale as the partnership pushed.

#### Synergy

Synergy refers to the positive impact or difference made through the partnership that could have otherwise not been achieved. The partnership created a platform for availability of professional scientific skills that resulted in more trained local staff on surgery and program implementation. At resource level, there were added permanent structures within the health system DP1 constructed two main clinics that could have otherwise not been. The table below reflects that through integration approach alone government would not reach the numbers that MOVE pushed for. The synergy produced can serve as a motivation to reinvest more resources on the program. The government is motivated to rethink strategy even though the partners had pulled away. As quoted above, the government would like to consider involving the community more (Table 3)

**Table 3:** Showing figures achieved through partnership

Year	Numbers reached
2007–2009	planning period
2009	5424 with integration only
2010	5773 with integration only
2011	14,661 with integration and MOVE
2012	38,005 with integration and MOVE
2013	46,793 with integration and MOVE
2014	30,033 with integration and MOVE

.

The target was to circumcise 100 thousand HIV negative men from 2012 till 2016. Figures confirmed with WHO [50] Progress Brief: Voluntary Medical Male Circumcision for HIV Prevention in 14 priority countries in East and Southern Africa.

#### Antagony

Antagony occurs when the partnership is viewed as a waste of time and resources; it is the outcome of partnership dysfunction where costs are more than benefits. When external donors felt they were spending more money but not achieving target they pulled away the DPs from the program without completing the mission.

## Discussion

### Input

The BMCF provides a framework for systematically examining the operationalisation of the partnership for SMC. The mission was to circumcise 80 % of HIV negative men in a 5 year period for HIV prevention. Corbin et al. ([33]: p.52) describe the mission as an “agreed-upon approach … to address a specific problem.” The partners were all working towards accomplishing the target of circumcising 80 % of HIV negative men by 2016. The first approach, integration, was a locally developed idea that they worked on together from the beginning; but the MOVE approach was externally formulated by PEPFAR as a complete package to implement with little or no flexibility. Concerning inputs MH contributed finance, health structures and medical equipment, and availed its medical staff for implementation. Both the development partners contributed human resources for capacity building and implementation of the program. The bulk of financial resources were reported to come from the DPs. Several authors on partnerships caution that resources offered by the North to the South deserve appreciation since they help bring change to some extent, regardless of being an instrument of control [4, 13, 24, 51].

The target which defined the mission caused challenges that ran through all components of partnership functioning, and caused failure to achieve the same. The target was not locally defined to include local realities, but was calculated using mathematical models and recommended by WHO. Waage et al. [35] recommend that it is important to ensure that targets that are set internationally are easy to translate nationally. All the three partners queried the target of circumcising 100 000 men per year. The expectation that the recipient would act in a certain predictable way was the greatest pit-fall of the mission [17]. Expected reciprocal response from communities, in the form of massive numbers circumcising did not materialise for different local reasons (see [52]). This is similar to what occurred with the millennium development goals that believe in creating targets to motivate but actually undermine the mission [35]. Respondents to Sjostedt’s study on aid effectiveness assessing NGOs using three different aid modalities in Tanzania, Cambodia and Zanzibar “..voiced concerns about whether or not the strict focus on results—and especially on reporting them—in fact channeled aid into easily measurable activities at the expense of more complex and long-term processes with potentially higher, but less easily measured, impacts.” ([36], p153). It is acknowledged that partners made strategic efforts to push the target but the same efforts also created conflict. For example, deployment of DPs’ staff to Government health centers and later separating them to form MOVE teams was appreciated as creating focus, yet also blamed for creating Government’s dependency on DPs. DHMTs’ priorities were ill patients. Waage et al. [35] observe that the focus on target rather than broader goals has contributed to countries distancing themselves from a global agenda that is seen as irrelevant in their particular development situation.

### Throughput

The functioning of the partnership is revealed through communication, input interaction, leadership, and partner roles. Each of the four elements shaping the collaborative context worked well at some stage in the partnership, but turned to be a source of contention through time. Although there were tensions between the in-country partners, they were working together in strategising to address some challenges of the partnership and implementation. Pressure from external donors undermined such efforts. Firstly, there was cooperation and agreement in the planning process where common implementation and reporting strategy were developed. However this did not live up to planned ideals at implementation level as DPs used different training tools, report tools and reported inconsistently to MH. Communication became a problem. Although in their studies, Weiss and colleagues [44] did not find that partner involvement challenges had any effect on synergy, they argue that less partner involvement in agreed strategies and goals, as well as lack of cooperation reduces synergy [44].

Secondly, the leadership of the program was clearly seen as MH’s. MH claimed and was referred to as a chair, coordinator and owner. Complementary to the leadership, DPs viewed themselves as ‘catalysts’ to help the government speed up the SMC scale up and as partners adding to resources necessary for successful implementation. However, the MH leadership role was questioned and contested through time. When MOVE superseded the integration approach, who owned and led the project became confused. WHO makes it clear that ownership of programs should be through participation of national leadership [5, 53]. The highest leadership of the country was criticized for not supporting the program as its “face” [24]. Members of Parliament reported being busy and having no capacity to participate. However, it is the political leadership that signed the MDGs and are rightfully challenged to show high participation [53]. Kenworthy [54] argues that recipient countries turn to endorse external new and exciting programs that they may not have the capacity to ‘own’ and sustain. It is very obvious that human resources for health in Botswana, although a factor beyond this partnership, need addressing. Human health resources is critical in combating all health challenges therefore national policies as well as global governance should consider that developing countries need additional heath workers to manage workload, since the workforce is already overloaded with multiple tasks [55, 56].

He adds that the fancy leadership/ownership phrases given the recipient countries are just a facade to cover the continuous exercise of power by the international financial agencies. Additionally DPs questioned the placement of MH national coordinators, suggesting change. They also queried DHMTs’ participation and leadership, questioning their priorities. Power and control of how things should work is in the nature of Northern partners [24]. In fact, DP1 confirmed that they brought in training on how the partnership and coordination should work. This goes with Abrahamsen’s observation that “..western countries and the institution of global governance still hold considerable sway over African states due primarily to their aid dependency and general economic weakness” [10]. Even though recipient countries are placed at the driver’s seat for driving the MDGs, critics observe that the recommended health programs often clash with in-country priorities [35].

Thirdly, there seemed to be clear roles and structure of the partnership. However the same also proved to be problematic. Ownership is defined by different leadership roles. Although all partners identified MH as owner, in practice ownership was measured according to the level of financial contribution. The DPs’ high level of funding mystified ownership and caused tension. Corbin et al. [33] contend that symbolic funding, (e.g MH’s contribution in terms of health structures and own employed staff) should have been seen as in-kind financial contribution [33]. However the same authors find this as a difficult issue because converting the cost of local in-kind contribution to dollars can be problematic. International donors (who were not ‘leading’ the partnership) controlled use of ‘donated’ financial resources. Whereas there seemed to be little transparency on the DPs’ use of funds, MH as ‘owner’ had to account for all program funds to OECD. MH also had to account for the imbalance in the usage of surgical equipment. The structure of the partnership seemed lopsided. In fact, as in most North-South partnerships, accountability seems to always be one-sided [13, 35]. This could also be explained by an observation of Corbin and Mittelmark [3] that partners may choose the route of blurry accountability if they experience that working mutually absorbs substantial resources and that consensus building procedures take a long time.

### Output and feed back to mission

Our findings show all levels of output in the BMFC: additive results through a revelation that MC was not a new thing that was brought in by the partnership, but that it has always been one of the health services given to the public. The partnership attained synergistic outcome as well, that is 2 + 2 = 5, where more results achieved through the partnership at integration and MOVE phases, which could have never been without the partnership. Types of synergy like shared knowledge between partners, shared resources and problem solving were evident in the partnership [43]. There was continued feedback into the mission throughout the throughput process as synergistic and antagonistic results were realised. Partners met annually to reflect on results and strategise on solutions. When the integration approach did not achieve sufficient output, in 2011 the partners agreed to strengthen the program by bringing in the MOVE approach. Although greater output was achieved through MOVE the contention on not meeting the target in 2012 resulted in antagony (DPs cutting down the financial and partners resources in 2013 and finally withdrawing in 2014). It is observed that external donors (PEPFAR and Bill and Melinda Gates Foundation) had greater influence on the functioning of the in-country partnership as vertical interaction (feedback) with them was demanded. This is a confirmation of the power of those holding the purse, the North, being greater than those with a begging bowl, the South [10]. The result was discord between in-country partners, with accusations on wasting time and money. Pressure from the Northern donors caused the DPs to withdraw from the partnership and the cost was a drastic decline in numbers of men coming for MC. The global context in which HIV/AIDS programs operate tends to be prescriptive, undermining locally initiated strategies and creating dependency [57]. Although the global health apparatus has an important context for developing interventions and mobilising resources for support, it cannot be successful without local participation. This implies that neither global nor national policies for health programs should be treated as absolute, but there has to be a genuine integration of indigenous and international strategies that if applied, could salvage massive resources that continue being lost as a result of vertical control [57, 58]. Also international organisations, like the DPs in this case, need to be given agency such that they play an advisory role to their international donors informing them of realities on the ground that cannot be overlooked.

According to the BMFC model antagony feeds in and out the collaborative process of the partnership and can be used positively to improve progress. Corbin et al. [33] argue that antagony can create an opportunity for partners to reflect on what went wrong and what could have been done differently. In this case, although left alone, MH was now reconsidering its whole approach, learning from the partnership mistakes and preparing to revisit and improve its integration approach to implementation. In fact, there has been debates over time on whether vertical programmes that attend to one problem at a time are effective or whether all health problems should be assimilated into the lager primary health care system, as the integration approach attempted [59]. The challenge of cost effectiveness is still to be addressed [59].

### Limitations

Whereas all partner organisations were given equal opportunities to participate in the research, the MH was more forthcoming in allowing several officers to be interviewed. MH was also more available for interviews at different phases as the partnership evolved. In that way some partners have not had a chance to give their full views and experiences at different phases of the partnership. However, each organisation had given its frank overall view of the partnership in the initial 3-day planning meeting that the researcher attended, and in immediate second interviews. Although it was evident that most decisions affecting implementation and partnership relations had an influence of the external donors’ pressures the first author was not able to access them for direct interviews. Systematic reflexivity was applied throughout the data collection and analysis stages. The results cannot be generalised to all countries, however they give important insightful lessons on partnership functioning for aspects that can be embraced and those that can be avoided. This paper does not look at factors that have led to particular outcomes, especially antagony or the nature of antagony, and this will be followed.

## Conclusion

This paper used a systems model to explore the functioning of the SMC partnership in Botswana. It has assessed different achievements and challenges that the partnership was facing. We conclude that external influences that come from the unseen international donors influenced the working of the in-country partnership, unfortunately crippling it from resolving implementation challenges as experienced within the context of partnership functioning. The experience of SMC partnership in Botswana showed that key influences on the success or failure of partnerships are financial resources, “ownership” and the target. The very mechanisms used for accountability by the Paris Declaration are sabotaged by the same global context where the exercise of power and financial leverage by international donors reign. A combination of inputs by partners brought progress towards achieving set program goals. However, prioritising externally formulated programs and lack of appreciation for local symbolic funding undermined local efforts and gave blurriness in leadership and ownership of the program. Pressure to meet the expectations of the international donors caused tension and challenges between the in-country partners and caused the DPs to retreat, and not pursue the mission further. Externally formulated goals and targets, as well as subsequent expectations from external donors placed the functioning and contextual interaction of the partnership at risk. Tensions in achieving the target, financial and in-kind resources and ownership queries resulted in DPs withdrawing before accomplishing the mission. All in all the key contribution of the study to the BMFC theory is that the functioning of the visible in-country partnership is significantly influenced by the less visible global context such as the target setters and donors.

## Abbreviations

ACHAP, Africa Comprehensive HIV/AIDS Partnership; BINAPS, Botswana National HIV/AIDS Prevention Support Project; CDC, Centers for Disease Control and Prevention; DHMT, District Health Management Team; MC, Medical Circumcision; MH, Ministry of Health; MOVE, Models for Optimizing Volume and Efficiency; OECD, Organization for Economic Cooperation and Development; PEPFAR, President’s Emergency Plan for AIDS Relief; PSI, Population Services International; SMC, Safe Male Circumcision; USAID, United States Agency for International Development; VMMC, Voluntary Medical Male Circumcision

## Footnotes

1 “Parental states” is not proper English, but is a direct translation from Setswana language phrase used by Government officers to show that the Government is the leader/the host /he owner/the one responsible for the program and other partners fall under their authority. That is why one of the officers referred to Government as a “parent” in one of the quotations below.
